# Outcomes of combined carbazochrome sodium sulfonate plus tranexamic acid therapy versus tranexamic acid monotherapy in traumatic brain injury: a retrospective cohort study in Japan

**DOI:** 10.1186/s40560-026-00855-w

**Published:** 2026-01-27

**Authors:** Jinsuke Mizuno, Yoshihisa Miyamoto, Yuichiro Matsuo, Kiyohide Fushimi, Ryota Inokuchi, Kent Doi, Hideo Yasunaga

**Affiliations:** 1 Department of Clinical Epidemiology and Health Economics, School of Public Health, The University of Tokyo, 7-3-1 Hongo, Bunkyo-Ku, Tokyo, Japan; 2 Department of Emergency and Critical Care Medicine, The University of Tokyo Hospital, Tokyo, Japan; 3 Department of Real-World Evidence, Graduate School of Medicine, The University of Tokyo, Tokyo, Japan; 4 Department of Health Policy and Informatics, Institute of Science Tokyo Graduate School, Tokyo, Japan

**Keywords:** Carbazochrome, Mortality, Tranexamic acid, Hemostasis, Traumatic brain injury, Intracranial hemorrhage

## Abstract

**Background:**

Traumatic brain injury (TBI) is a major public health concern associated with substantial morbidity and mortality. In Japan, carbazochrome sodium sulfonate (CSS) is widely used, often in combination with tranexamic acid (TXA), for the management of various types of bleeding; however, studies on the effectiveness of CSS in TBI are scarce. Therefore, this study aimed to investigate the association between the use of CSS plus TXA versus TXA alone and the clinical outcomes in patients with TBI.

**Methods:**

This observational study was conducted using data retrieved from the Japanese Diagnosis Procedure Combination database between July 2010 and March 2022. We enrolled adult patients aged ≥ 16 years diagnosed with TBI who received TXA on the day of admission. Patients with chronic subdural hematoma, suspected TBI diagnosis, or severe extracranial trauma were excluded. The exposure was CSS plus TXA administration on the day of admission, with TXA monotherapy assigned as the control. The primary outcome was 28-day in-hospital mortality, and the secondary outcomes were 7-day in-hospital mortality, overall in-hospital mortality, consciousness at discharge, and length of hospital stay. We used propensity-score overlap weighting to balance patient characteristics between the groups.

**Results:**

This study included 150,026 patients. Of these, 17,212 (11.5%) received TXA alone, and 132,814 (88.5%) received CSS plus TXA. After propensity score overlap weighting, the primary outcome did not differ significantly between the TXA-only and CSS plus TXA groups (11.7% vs. 11.9%; risk difference, 0.1%; 95% CI − 0.4 to 0.7%). The secondary outcomes were also comparable between the two groups. However, the subgroup analysis restricted to unarousable patients (Japan Coma Scale 100–300) revealed a significant reduction in the 7-day mortality in the CSS plus TXA group.

**Conclusions:**

Combined treatment with CSS and TXA was not associated with better clinical outcomes in terms of in-hospital mortality, consciousness at discharge, or length of hospital stay in hospitalized adult patients with TBI compared with TXA therapy alone. Routine use of CSS may not be recommended.

**Supplementary Information:**

The online version contains supplementary material available at 10.1186/s40560-026-00855-w.

## Background

Traumatic brain injury (TBI) is a major public health concern associated with substantial morbidity and mortality [1–3]. Secondary brain injury resulting from progressive intracranial hemorrhage (ICH), hematoma, and cerebral edema is an extremely serious complication of TBI, whose expansion can worsen the patient’s condition [4]. These conditions often require invasive surgeries and prolonged hospital stay that can compromise the patient’s quality of life or even result in fatality [5].

Although the current guidelines for TBI in the United States and Japan focus on decompressive craniectomy, therapeutic hypothermia, and osmotic drugs, evidence on the effectiveness of hemostatic drugs for preventing hematoma expansion has not been established [6, 7]. Recently, intensive investigations into the protective effects of tranexamic acid (TXA) have demonstrated an improvement in mortality in cases of severe trauma (CRASH-2 study [8]) and mild traumatic ICH (CRASH-3 study [9]). In addition to these major studies, the efficacy of TXA in TBI has been widely evaluated [10].

Carbazochrome sodium sulfonate (CSS) is another hemostatic agent introduced in the late 1940s [11, 12]. CSS is a stabilized metabolite of adrenochrome semicarbazone (an oxidized metabolite of adrenaline). Its hemostatic effect is primarily ascribed to the reduction in capillary permeability and increase in capillary resistance [13]. Another possible molecular mechanism involves inhibition of phosphatidylinositol hydrolysis in the vascular endothelial cells [14]. However, many studies have suggested that the direct effect of CSS on platelet aggregation and blood coagulation cascade is limited or non-existent [13, 14].

Currently, CSS is approved in countries such as Japan, China, Indonesia, and Egypt, but remains unapproved in the United States and most European countries. Since the 1960s, CSS has been commonly used in Japan, often in combination with TXA, for the management of a broad range of hemorrhagic conditions, including ICH, gastrointestinal bleeding, hemorrhoids, and hemoptysis [15–18], but the few studies conducted on its effectiveness have reported inconsistent results. According to single-center, observational studies, CSS use was not associated with an improved postprocedural bleeding rate (*n* = 304) [19] and lower mortality in patients with bleeding trauma (*n* = 259) [20]. A randomized controlled trial (*n* = 150) found that CSS reduced bleeding during hip replacement surgery [21], while a cohort study (*n* = 10) found that it controlled epistaxis in Osler’s disease [22]. However, the above-mentioned studies incorporated small sample populations, and none focused on TBI. Hypothetically, administering CSS in addition to TXA promotes capillary constriction, thereby leading to enhanced hemostasis and improved clinical outcomes in patients with TBI.

Therefore, we aimed to investigate the association of CSS plus TXA therapy versus TXA monotherapy with the outcomes in patients with TBI by analyzing large-scale data from the Diagnosis Procedure Combination (DPC) database. We compared TXA plus CSS with TXA monotherapy rather than comparing CSS with no-hemostats control for two reasons. First, TXA is already commonly used for hemostasis after TBI in Japan [23]. Second, the absence of hemostats may reflect a decision to limit care intensity, which would introduce significant selection bias.

## Methods

### Data source

This observational study was conducted using data retrieved from the Japanese DPC database [24]. The DPC database comprises discharge summaries and administrative claims data from voluntarily participating hospitals, representing over 1500 acute-care hospitals and encompassing approximately 50% of all beds from the latter [25]. It is a repository of patient demographics for all hospitalizations such as age, sex, and smoking history; diagnoses coded using the International Classification of Diseases, Tenth Revision (ICD-10); daily procedures recorded with the Japanese procedural codes; daily drug administration; and admission and discharge status. Interventions performed in the emergency department before hospital admission were designated as being performed on day 1 (the day of admission) when the patients were admitted through the emergency department. Pre-hospital administration of hemostatic agents is not performed in Japan. A previous validation study demonstrated high specificity and moderate sensitivity for the recorded diagnoses as well as high specificity and sensitivity for the recorded procedures [26]. This was a retrospective observational study and was not registered prospectively since trial registration is not applicable to this type of study.

### Patient selection

We identified adult patients aged ≥ 16 years who were diagnosed with TBI (ICD codes: S061, S062, S063, S064, S065, S066, and S068; Supplemental Table 1) and treated with TXA on the day of admission between July 2010 and March 2022 from the database. Of note, the database utilizes the ICD-10 codes based on the Japanese standards issued in 2003 and revised in 2013, rather than ICD-10-CM. The revision in 2013 did not affect codes utilized in this study. Only the first admission was considered for patients who were admitted to the hospital more than once with a diagnosis of TBI. We excluded patients who fulfilled the following criteria: diagnosis of chronic subdural hematoma alone by text searching in the written Japanese diagnosis given along with the ICD-10 codes to focus on acute traumatic ICH since S065 includes both acute and chronic subdural hematoma; suspected diagnosis of TBI; and comorbid diagnosis of severe extracranial trauma according to the method described in the subsequent subsection.

### Assessment of extracranial trauma severity

The Abbreviated Injury Scale (AIS) and Injury Severity Score (ISS) were used to quantify the severity of injury [27]. The AIS grades individual injuries into six severity levels ranging from 1 (minor) to 6 (unsurvivable). The ISS aims to determine the injury severity of each patient with single or multiple traumas by summing the squares of the AIS scores from the three most severely injured anatomical body regions. A patient with any injury rated as 6 on the AIS is automatically assigned a maximum ISS of 75. To identify patients with significant extracranial trauma, we first converted the ICD-10 codes to the AIS scores and their corresponding six anatomical body regions using the ICD-PIC-R data Table [28, 29] To make this data table suitable for the Japanese context, we converted the ICD-10 CM codes from the original table into the corresponding ICD-10 codes used in Japan by matching both code sets up to the fifth digit. Subsequently, we excluded patients whose converted AIS score was 3 or higher for any body region, except for the head, because an AIS of 3 (severe, but not life-threatening) is a widely accepted criterion for severe trauma [30]. Furthermore, we excluded patients identified with unique Japanese comorbid ICD-10 codes that indicated trauma accompanied by vascular and/or thoracoabdominal organ injuries (Supplemental Table 2). This methodological approach was adopted from a previous study that focused on predicting trauma-related mortality using modified Japanese ICD-10 codes [31].

### Exposure and outcomes

The exposure was co-administration of CSS and TXA on day 1 (the admission day), with TXA monotherapy on day 1 assigned as the control group. The primary outcome was 28-day in-hospital mortality. The secondary outcomes included 7-day in-hospital mortality, overall in-hospital mortality, consciousness at discharge, and length of hospital stay. The level of consciousness at discharge was categorized using the four-point Japan Coma Scale (JCS): alert (0), awake without stimulation (1-digit: 1–3), arousable with stimulation (2-digit: 10–30), and unarousable (3-digit: 100–300). These categories demonstrate strong predictive accuracy for in-hospital mortality in patients with trauma and correspond well with the Glasgow Coma Scale (GCS)-based severity classification of mild (GCS 13–15), moderate (GCS 9–12), and severe (GCS 3–8) TBI.

### Covariates

Based on previous studies [32–34] and clinical relevance, we included covariates from the following three main categories: patient characteristics at admission, treatment and procedures on day 1, and institutional and contextual characteristics. Patient characteristics at admission included age, sex, body mass index (BMI), smoking status (categorized as nonsmoker, current, or former smoker), and comorbidities as defined by the Charlson Comorbidity Index (CCI) [35]. BMI was classified according to the World Health Organization criteria for the Asian population [36]: less than 18.5, 18.5–22.9, 23.0–24.9, and 25.0 kg/m^2^ or more. We also included the head AIS score, ISS, injury type (traumatic cerebral edema, diffuse brain injury, focal brain hemorrhage, epidural hemorrhage, subdural hemorrhage, subarachnoid hemorrhage, and other specified intracranial injuries), and the JCS score at admission [37]. The JCS score at admission was treated as a granular categorical variable with 10 distinct levels to allow for the most rigorous adjustment possible. Treatment and procedures on day 1 included neurosurgery, noninvasive oxygen administration, intubation, dialysis, vasopressors, transfusion of fresh-frozen plasma and platelet concentrate, and anticoagulant-antagonist. Vasopressor use was regarded as a proxy for hypotension, transfusion of fresh-frozen plasma or platelet concentrate for coagulopathy, and anticoagulant-antagonist for anticoagulant medication, respectively. Details on neurosurgery are provided in Supplemental Table 3.

Institutional and contextual characteristics included fiscal year, season, TBI-related hospital volume, ambulatory transport, night/holiday admission, admission ward type (general ward; intensive care unit [ICU]; high-dependency unit [HDU]; stroke care unit [SCU]), and university hospital or non-university hospital. Seasons were categorized into March–May, June–August, September–November, and December–February. The TBI-related hospital volume was categorized by the number of annual admissions for the study cohort: 1–30, 31–60, and > 60 cases.

### Statistical analysis

Missing values for BMI, smoking status, and ambulatory transport were imputed using multiple imputations with chained equations, and 20 sets of imputed data were created [38]. We used propensity score overlap weighting to account for potential confounding factors between the CSS plus TXA and TXA-only groups. The propensity score for the receipt of CSS plus TXA therapy was estimated by using a multivariable logistic regression model, which incorporated all the covariates mentioned earlier as independent variables. Overlap weighting was conducted using propensity scores. The overlap weighting method balances the treatment and control groups by assigning weights to each patient based on their probability of receiving the opposite treatment [39, 40]. As a result, individuals with a similar propensity to receive either of the two treatment regimens (viz. CSS plus TXA or TXA only) are assigned greater weights, whereas those with a lower propensity to receive the alternative treatment are assigned smaller weights. To evaluate balance, we calculated the absolute standardized mean differences (ASDs) for each variable between the two groups. An ASD value exceeding 10% indicates a significant imbalance. Finally, we conducted generalized linear regression analyses for the binomial outcomes to calculate the risk difference (RD) with its 95% confidence interval (CI) between the two treatment groups. We conducted proportional ordinal logistic regression to assess the distribution of the JCS scores at discharge and quartile regression for the length of hospital stay. We also performed a subgroup analysis according to the level of consciousness at admission: alert (JCS 0), awake (JCS 1–3), arousable (JCS 10–30), and unarousable (JCS 100–300). We also performed several sensitivity analyses. First, we performed a complete case analysis of missing values of smoking history and BMI. Second, we performed another analysis by adjusting for neurosurgery, intracranial pressure (ICP) monitoring, and administration of antihypertensive agents and osmotic agents on day 1. We excluded these interventions from the covariates in the main analysis because they could act as mediating factors in the pathway between the treatment and outcome. All statistical analyses were performed using STATA/SE version 19.0 (Stata Corp, College Station, TX, USA).

## Results

**Fig. 1:**
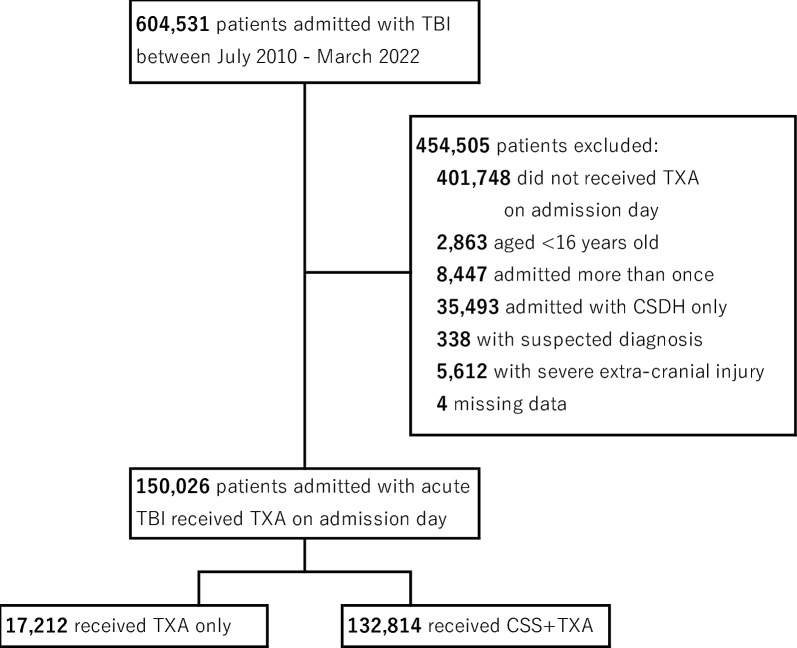
Patient selection flow diagram. TBI, traumatic brain injury; TXA, tranexamic acid; CSDH, chronic subdural hematoma; CSS, carbazochrome sodium sulfonate

A total of 150,026 adult patients with acute TBI were enrolled in this study. Of them, 11.5% (17,212/150,026) received only TXA and 88.5% (132,814/150,026) received CSS plus TXA (Fig. 1).

Table 1 summarizes the baseline characteristics of the cohorts before and after propensity score overlap weighting (only major covariates are shown due to space limitation; the complete table is provided in Supplemental Table 4). In the unweighted cohort, patients in the CSS plus TXA group were less likely to be intubated or receive noradrenaline, fresh-frozen plasma, and platelets than those in the TXA-only group. TXA monotherapy was delivered more frequently in recent years, in teaching and university hospitals, when ambulances were used for patient transport, and less frequently on weekends or night admissions. Comorbidities and diagnosis of the type of head injury were not associated with treatment selection. After propensity score overlap weighting, the baseline characteristics appeared to be well balanced between the two groups.

**Table 1:** Baseline patient characteristics before and after propensity score overlap weighting (summarized version)

	Before weighting					After PS overlap weighting		
	TXA only		CSS plus TXA		ASD	TXA only	CSS plus TXA	ASD
Variable	(*n* = 17,212)		(*n* = 132,814)					
Age, mean (SD)	71.7	(17.2)	73.9	(15.6)	0.13	72.4 (16.7)	72.4 (16.6)	0.00
Male	10,584	(61%)	78,748	(59%)	0.05	(61%)	(61%)	0.00
Body mass index								
< 18.5	2774	(16%)	23,906	(18%)	0.05	(17%)	(17%)	0.00
18.5–23	8029	(47%)	63,521	(48%)	0.02	(47%)	(47%)	0.00
23–25	3081	(18%)	23,049	(17%)	0.01	(18%)	(18%)	0.00
> 25	3328	(19%)	22,338	(17%)	0.07	(19%)	(19%)	0.00
Current/past-smoker	4938	(29%)	36,675	(28%)	0.02	(29%)	(29%)	0.00
Comorbidities								
Congestive heart failure	535	(3%)	5284	(4%)	0.05	(3%)	(3%)	0.00
Cerebrovascular disease	1084	(6%)	10,749	(8%)	0.07	(7%)	(7%)	0.00
Severe liver disease	2180	(13%)	18,525	(14%)	0.04	(13%)	(13%)	0.00
Diabetes	323	(2%)	2428	(2%)	0.00	(2%)	(2%)	0.00
Renal disease	475	(3%)	4328	(3%)	0.03	(3%)	(3%)	0.00
AIS of the head, mean (SD)	1.8	(0.8)	1.7	(0.7)	0.06	1.8 (0.8)	1.8 (0.8)	0.00
ISS, mean (SD)	4.4	(4.1)	3.9	(3.3)	0.14	4.2 (3.7)	4.2 (4.1)	0.00
Details of head injury								
Brain edema	16	(0.1%)	85	(0.1%)	0.01	(0.1%)	(0.1%)	0.00
DAI	3309	(19%)	22,853	(17%)	0.05	(19%)	(19%)	0.00
Focal hematoma	64	(0.4%)	482	(0.4%)	0.00	(0.4%)	(0.4%)	0.00
AEDH	85	(0.5%)	498	(0.4%)	0.02	(0.5%)	(0.5%)	0.00
ASDH	7747	(45%)	64,684	(49%)	0.07	(46%)	(46%)	0.00
Traumatic SAH	6517	(38%)	45,541	(34%)	0.07	(37%)	(37%)	0.00
Others	460	(3%)	4370	(3%)	0.04	(3%)	(3%)	0.00
Japan Coma Scale								
0 (alert)	4082	(24%)	37,718	(28%)	0.11	(25%)	(25%)	0.00
1–3 (awake)	6786	(39%)	59,850	(45%)	0.11	(41%)	(41%)	0.00
10–30 (arousable)	2424	(14%)	17,128	(13%)	0.04	(14%)	(14%)	0.00
100–300 (unarousable)	3920	(23%)	18,118	(14%)	0.24	(20%)	(20%)	0.00
Intubation	2861	(17%)	7886	(6%)	0.34	(13%)	(13%)	0.00
Noradrenaline	1042	(6%)	2808	(2%)	0.20	(5%)	(5%)	0.00
FFP	2100	(12%)	5999	(5%)	0.28	(9%)	(9%)	0.00
Platelet	625	(4%)	2529	(2%)	0.11	(3%)	(3%)	0.00
Menatetrenone	725	(4%)	6949	(5%)	0.05	(4%)	(4%)	0.00
Fiscal year								
2010–2014^†^	3734	(22%)	47,244	(36%)	0.31	(24%)	(24%)	0.00
2015–2018^†^	5470	(32%)	46,306	(35%)	0.07	(33%)	(33%)	0.00
2019–2022^†^	8008	(47%)	39,264	(30%)	0.36	(44%)	(44%)	0.00
TBI-related hospital volume, per year								
< 31	11,023	(64%)	75,990	(57%)	0.14	(62%)	(62%)	0.00
31–60	4783	(28%)	42,284	(32%)	0.09	(29%)	(29%)	0.00
> 60	1406	(8%)	14,540	(11%)	0.10	(9%)	(9%)	0.00
Admission ward								
ICU	4757	(28%)	22,414	(17%)	0.26	(24%)	(24%)	0.00
HDU	5714	(33%)	39,083	(29%)	0.08	(33%)	(33%)	0.00
University hospital	3054	(18%)	10,386	(8%)	0.30	(15%)	(15%)	0.00

Reported as n (%) or (%), unless indicated otherwise. The absolute standardized difference (ASD) was calculated after multiple imputations. †The Japan Coma Scale score at admission and the fiscal year were categorized due to space limitations. The SMD for the categorized covariates was separately calculated for the table. A complete version of the baseline characteristics is provided in the supplemental information (Supplemental Table 4)

SD, standard deviation; AIS, Abbreviated Injury Scale; ISS, injury severity score; DAI, diffuse axonal injury; AEDH, acute epidural hemorrhage; ASDH, acute subdural hemorrhage; SAH, subarachnoid hemorrhage; FFP, fresh-frozen plasma; TBI, traumatic brain injury; ICU, intensive care unit; HDU, high-dependency unit

Table 2 shows the outcomes after propensity score overlap weighting. The primary outcome, i.e., the 28-day in-hospital mortality, was 11.7% and 11.9% in the TXA only and CSS plus TXA groups, respectively [RD, 0.1; 95% CI, -0.4 – 0.7%]. The 7-day in-hospital mortality, overall in-hospital mortality consciousness at discharge, and length of hospital stay were also similar between the two groups.

**Table 2:** Comparison of clinical outcomes between the TXA-only and CSS plus TXA groups after propensity score overlap weighting

	Overlap weighted cohort		
Outcomes	TXA only	CSS plus TXA	Risk difference or effect size (95% CI)
Primary outcome			
28-day in-hospital mortality	11.7%	11.8%	0.1% (− 0.4% to 0.7%)
Secondary outcome			
7-day in-hospital mortality	7.9%	7.6%	− 0.4% (− 0.8% to 0.7%)
In-hospital mortality	13.1%	13.6%	0.5% (− 0.0% to 1.1%)
Consciousness at discharge			
JCS 0 (alert)	54.7%	54.9%	1.01 (0.96 to 1.05)
JCS 1–3 (awake)	27.7%	26.7%	
JCS 10–30 (arousable)	2.7%	3.1%	
JCS 100–300 (unarousable)	1.9%	1.8%	
Length of hospital stay, days, median (IQR)	14 (6–31)	15(6–34)	1 (− 0.2 to 2.2)

For in-hospital mortality, the risk difference was calculated using a generalized linear regression model to compare the event probability in the CSS plus TXA group relative to the TXA-only group. Ordinal logistic regression was used to calculate the proportional odds ratio for consciousness at discharge, comparing the odds of being in a higher category for the CSS plus TXA group relative to the TXA-only group. For the length of hospital stay, the difference in the median was calculated using a median regression model to compare the median in the CSS plus TXA group versus the TXA-only group. All analyses were conducted after propensity score overlap weighting and multiple imputations for missing values

TXA, tranexamic acid; CSS, carbazochrome sodium sulfonate; JCS, Japan Coma Scale; IQR, interquartile range

Table 3 shows the results of the subgroup analysis according to the level of consciousness at admission. In the unarousable patient subgroup, the 7-day in-hospital mortality was significantly lower in the CSS plus TXA group [32.4% in TXA only vs. 29.5% in CSS plus TXA group, RD -2.8% (95% CI -4.6% to -1.1%)], whereas the 28-day and total in-hospital mortality did not differ significantly between the treatment groups. Consciousness at discharge and the length of hospital stay were similar between the CSS plus TXA and TXA-alone groups. The results of sensitivity analyses (Table 4) approximated those of the main analysis. Before overlap weighting, neurosurgery on day 1 was performed in 13% (2,284/17,212) of patients in the TXA group and 10% (13,764/132,814) in the CSS plus TXA group, whereas after overlap weighting, the proportion was 12% in both groups (Supplemental Table 6).

**Table 3:** Comparison of clinical outcomes in subgroups by the Japan Coma Scale score at admission

		Overlap weighted cohort		
Outcomes	Subgroups	TXA only	CSS plus TXA	Risk difference or Effect size (95% CI)
Primary outcome				
28-day in-hospital mortality	JCS 0	2.5%	2.8%	0.3% (− 0.2% to 0.8%)
	JCS 1–3	3.8%	4.1%	0.2% (− 0.3% to 0.7%)
	JCS 10–30	8.0%	9.0%	1.1% (− 0.1% to 2.3%)
	JCS 100–300	42.4%	41.2%	− 1.2% (− 3.0% to 0.7%)
Secondary outcome				
7-day in-hospital mortality	JCS 0	1.2%	1.5%	0.4% (0.0% to 0.7%)
	JCS 1–3	1.6%	1.8%	0.2% (− 0.2% to 0.5%)
	JCS 10–30	4.1%	4.3%	0.3% (− 0.6% to 1.1%)
	JCS 100–300	32.4%	29.5%	− 2.8% (− 4.6% to − 1.1%)
In-hospital mortality	JCS 0	3.1%	3.7%	0.6% (0.0% to 1.2%)
	JCS 1–3	4.9%	5.6%	0.7% (0.1% to 1.7%)
	JCS 10–30	9.7%	11.5%	1.8% (0.4% to 3.1%)
	JCS 100–300	45.1%	44.1%	− 1.0% (− 2.8% to 0.9%)
Consciousness at discharge				
JCS0 (alert)	JCS 0	87.2%	88.2%	0.87 (0.76 to 1.00)
JCS 1–3 (awake)		9.1%	7.6%	
JCS 10–30 (arousable)		0.4%	0.4%	
JCS 100–300 (unarousable)		0.2%	0.2%	
JCS0 (alert)	JCS 1–3	54.0%	54.3%	0.98 (0.91 to 1.05)
JCS 1–3 (awake)		39.4%	38.3%	
JCS 10–30 (arousable)		1.2%	1.5%	
JCS 100–300 (unarousable)		0.5%	0.3%	
JCS0 (alert)	JCS 10–30	48.1%	47.4%	1.12 (1.00 to 1.26)
JCS 1–3 (awake)		34.9%	33.9%	
JCS 10–30 (arousable)		5.8%	6.2%	
JCS 100–300 (unarousable)		1.4%	1.0%	
JCS0 (alert)	JCS 100–300	19.6%	19.3%	1.00 (0.91 to 1.10)
JCS 1–3 (awake)		21.4%	22.2%	
JCS 10–30 (arousable)		6.6%	7.3%	
JCS 100–300 (unarousable)		7.2%	7.1%	
Length of hospital stay, median (IQR)	JCS 0	9 (4–20)	9 (4–21)	0 (− 1.3 to 1.3)
	JCS 1–3	14 (7–29)	15 (6–31)	1 (− 0.6 to 2.6)
	JCS 10–30	23 (10–39)	24 (11–43)	1 (− 2.8 to 4.8)
	JCS 100–300	18 (3–43)	21(4–48)	3 (− 1.4 to 7.4)

We conducted subgroup analysis stratified by consciousness at admission. For in-hospital mortality, the risk difference was calculated using a generalized linear regression model to compare the event probability in the CSS plus TXA group relative to the TXA-only group. Ordinal logistic regression was used to calculate the proportional odds ratio for consciousness at discharge, comparing the odds of being in a higher category for the CSS plus TXA group relative to the TXA-only group. For the length of hospital stay, the difference in the median was calculated using a median regression model to compare the median in the CSS plus TXA group relative to the TXA-only group. All analyses were conducted after propensity score overlap weighting and multiple imputations for missing values

TXA, tranexamic acid; CSS, carbazochrome sodium sulfonate; JCS, Japan Coma Scale; IQR, interquartile range

**Table 4:** Results of sensitivity analysis for the clinical outcomes, including a complete case analysis and an analysis with further adjustment for potential mediators (intracranial pressure monitoring, neurosurgery, and administration of antihypertensive agents and osmotic agents on day 1)

Complete cases analysis	Overlap weighted cohort		
Outcomes	TXA only	CSS plus TXA	Risk difference or effect size (95% CI)
Primary outcome			
28-day in-hospital mortality	9.0%	9.2%	0.2% (− 0.4% to 0.7%)
Secondary outcome			
7-day in-hospital mortality	5.6%	5.4%	− 0.2% (− 0.6% to 0.3%)
In-hospital mortality	10.4%	10.9%	0.6% (− 0.0% to 1.1%)
Consciousness at discharge			
JCS 0 (alert)	57.0%	57.4%	1.00 (0.95 to 1.06)
JCS 1–3 (awake)	28.6%	27.1%	
JCS 10–30 (arousable)	2.5%	3.0%	
JCS 100–300 (unarousable)	1.6%	1.6%	
Length of hospital stay, median (IQR)	15 (6–32)	15 (6–35)	0 (− 1.0 to 1.0)
Further adjustment for possible mediators	Overlap weighted cohort		
Primary outcome			
28-day in-hospital mortality	11.6%	11.7%	0.1% (− 0.4 to 0.7%)
Secondary outcome			
7-day in-hospital mortality	7.8%	7.6%	− 0.2% (− 0.7% to 0.2%)
In-hospital mortality	12.9%	13.5%	0.5% (− 0.1% to 1.1%)
Consciousness at discharge			
JCS 0 (alert)	54.8%	55.4%	0.99 (0.95 to 1.04)
JCS 1–3 (awake)	27.7%	26.5%	
JCS 10–30 (arousable)	2.7%	3.0%	
JCS 100–300 (unarousable)	1.9%	1.7%	
Length of hospital stay, median (IQR)	14 (6–31)	14 (6–33)	1 (− 1.2 to 1.2)

The following sensitivity analyses were conducted: we performed complete cases analysis for missing values (body mass index, smoking history, and ambulance use); we further adjusted for potential mediators (intracranial pressure monitoring, neurosurgery, and administration of antihypertensive agents and osmotic agents on day 1) in propensity score overlap weighting. For in-hospital mortality, the risk difference was calculated using a generalized linear regression model to compare the event probability in the CSS plus TXA group relative to the TXA-only group. Ordinal logistic regression was used to calculate the proportional odds ratio for consciousness at discharge, comparing the odds of being in a higher category for the CSS plus TXA group relative to the TXA-only group. For the length of hospital stay, the difference in the median was calculated using a median regression model to compare the median in the CSS plus TXA group relative to the TXA-only group.

TXA, tranexamic acid; CSS, carbazochrome sodium sulfonate; JCS, Japan Coma Scale; IQR, interquartile range

## Discussion

Using a nationwide claims database, we found that the addition of CSS to TXA was not associated with improvements in in-hospital mortality in hospitalized adult patients with TBI. Consistent with this finding, there was no significant association with consciousness level at discharge or length of hospital stay, which we included as a proxy for post-discharge prognosis given that the DPC database does not provide follow-up data after discharge. It may be worth noting that the 7-day in-hospital mortality in unarousable patients was significantly lower in the CSS plus TXA group.

While previous studies have demonstrated meaningful differences in the intermediate outcomes (e.g., 5-unit difference in the packed red blood cell transfusion volume in trauma-related bleeding [41] and 200–500 mL difference in intraoperative blood loss during arthroplasty [21, 42, 43]), the difference in mortality reported by the other two studies investigating CSS use in trauma-related bleeding lacked statistical significance [20, 41]. Therefore, the absence of an overall benefit in our study is consistent with prior findings.

Although CSS is expected to promote hemostasis via reduced capillary permeability and vasoconstriction, our results showed no association between CSS and TBI-related in-hospital mortality. Two different hypotheses may explain this null finding. The first possible explanation involves a trade-off on mortality between hemostasis and perfusion. Previous trials in ICH have highlighted that hemostatic efficacy does not automatically translate to improved clinical outcomes [44, 45]. For instance, the FAST trial (recombinant activated factor VII) [45] and the TICH-2 trial (TXA) [44] demonstrated that while these agents reduced hematoma growth, they failed to improve functional outcomes, partly due to ischemic complications or other competing risks. Hypothetically, while CSS-induced vasoconstriction helps control bleeding, it may simultaneously decrease cerebral blood flow. This potential exacerbation of ischemia could cancel out the beneficial hemostatic effects of CSS on mortality. The other hypothesis involves the unique physiology of the cranium. In the closed intracranial space, hematoma expansion following TBI increases ICP, naturally leading to a tamponade effect that inhibits further bleeding [46, 47]. A retrospective study has reported that brain atrophy—which implies a reduced tamponade effect—was associated with hematoma expansion in ICH, particularly in the presence of coagulopathy [48]. This finding suggests that the tamponade effect likely plays a synergistic role with the coagulation system in maintaining hemostatic balance. Therefore, especially in severe cases where the physical tamponade effect is sufficiently operative, the added vasoconstrictive effect of CSS may offer little additional survival benefit. Moreover, our findings should be interpreted within the context of concurrent TXA use. When CSS is used in conjunction with TXA, the additional effect of CSS-induced vasoconstriction and reduced permeability beyond intracranial tamponade may be small, particularly when fibrinolysis-driven hematoma expansion—a major cause of early deterioration in TBI [49]—is already targeted by TXA.

Interestingly, our subgroup analysis showed that CSS co-administered with TXA yielded a small but significant short-term reduction in mortality among patients with JCS scores of 100–300 (unarousable). In such severe TBI, massive ICH may overwhelm the tamponade effect, particularly in the presence of excessive fibrinolysis and microvascular injury. In such situations, CSS may offer advantages by decreasing vascular permeability and promoting vasoconstriction [8, 9]. This could potentially be an advantage unique to CSS, conferred by a hemostatic mechanism that is distinct from that of TXA. However, the reduction in short-term mortality among unarousable patients should be interpreted with caution. The lack of difference in 28-day in-hospital mortality suggests that the initial survival benefit may have been offset by the severity of the primary injury or subsequent withdrawal of life-sustaining measures. Therefore, results of 7-day in-hospital mortality should be considered exploratory and hypothesis-generating. Further pharmacological investigations delving into the role of CSS in TBI that account for varying injury severities are expected to address this issue. The clinical relevance of CSS that extends beyond the reduction of mortality to the reduction of hematoma expansion and the subsequent need for surgical intervention may be of interest to future researchers.

Some limitations of this study should be acknowledged. First, this was a retrospective database study, and unmeasured confounders such as the mechanism of injury, exact timing of drug administration, pupillary findings, baseline prescriptions, and functional status may have influenced the results. Although we rigorously adjusted for available proxies such as reversal agents for anticoagulants and tracheal intubation for herniation signs, residual confounding cannot be fully excluded. Details of the drug regimen (e.g., dosage and method of administration) were not assessed. Although a study on endoscopic procedural bleeding did not suggest a dose-dependent difference in the hemostatic effect of CSS [50], existing evidence regarding CSS regimens in patients with TBI is scarce. Therefore, the optimal dosage and administration methods of CSS for TBI require further investigation in future studies. Second, some factors such as osmotic agents, antihypertensives, and neurosurgery were not included in the propensity score overlap weighting. As the DPC database does not provide the intraday treatment timing, we could not determine whether these agents functioned as confounders or mediators. To overcome this issue, we performed a sensitivity analysis adjusted for ICP monitoring, neurosurgery, and administration of antihypertensive agents and osmotic agents on day 1, which yielded results similar to those of the main analysis. Third, misclassification in ICD-10 diagnoses and extracranial injury severity from ICD to AIS mapping may have obscured the associations. Fourth, the specific cause of death could not be assessed due to the lack of coding in the database, restricting clinical interpretation. Fifth, the specific etiology of impaired consciousness at admission (e.g., post-traumatic seizures) could not be determined. Although it is unlikely that the decision to administer CSS differed systematically between groups, given the lack of seizure-related contraindications, residual confounding by unmeasured pathophysiological conditions cannot be fully excluded.

## Conclusions

The co-administration of CSS with TXA was not associated with better clinical outcomes in terms of in-hospital mortality, consciousness at discharge, and length of stay in hospitalized adult patients with TBI compared with TXA monotherapy. Therefore, the routine use of CSS may not be recommended. While exploratory analyses suggested a potential short-term survival benefit among patients with severe consciousness impairment, these findings are hypothesis-generating. Future prospective studies are needed to validate these results and should ideally include assessment of intermediate outcomes, such as hematoma expansion and surgical interventions, to fully elucidate the clinical utility of CSS.

## Supplementary Information

Supplementary Material 1.

## Data Availability

The datasets used and/or analyzed during the current study are available from the corresponding author on reasonable request.

## Abbreviations

TBI: Traumatic brain injury
TXA: Tranexamic acid
CSS: Carbazochrome sodium sulfonate
DPC: Diagnosis procedure combination
ICD-10: International Classification of Diseases, Tenth Revision
AIS: The Abbreviated Injury Scale
ISS: Injury Severity Score
BMI: Body mass index
CCI: Charlson comorbidity index
JCS: Japan Coma Scale
GCS: Glasgow Coma Scale
ICU: Intensive care unit
HDU: High-dependency unit
SCU: Stroke care unit
ASD: Absolute standardized mean difference
RD: Risk difference
CI: Confidence interval
ICP: Intracranial pressure
ICH: Intracranial hemorrhage

## References

[1] Capizzi A, Woo J, Verduzco-Gutierrez M. Traumatic brain injury: an overview of epidemiology, pathophysiology, and medical management. Med Clin North Am. 2020;104:213–38. 32035565 10.1016/j.mcna.2019.11.001

[2] Facts about TBIU.S. Centers for disease control and prevention. 2025.

[3] The Japanese Association for the Surgery of Trauma, (Trauma Registry Committee), the Japanese Association for Acute Medicine, (Committee for Clinical Care Evaluation). Japan Trauma Data Bank Report 2020. 2020.

[4] Abda M, Needham K, Shakur-Still H, et al. Tranexamic acid in traumatic brain injury: an explanatory study nested within the CRASH-3 trial. Eur J Trauma Emerg Surg. 2021;47:261–8. 32076783 10.1007/s00068-020-01316-1 PMC7851008

[5] Kowalski RG, Hammond FM, Weintraub AH, et al. Recovery of consciousness and functional outcome in moderate and severe traumatic brain injury. JAMA Neurol. 2021;78:548–57. 33646273 10.1001/jamaneurol.2021.0084 PMC7922241

[6] The Japan Neurosurgical Society, the Japan Society of Neurotraumatology. Guidelines for the Management of Traumatic Brain Injury 4th Edition. Tokyo: IGAKU-SHOIN; 2019.

[7] Carney N, Totten AM, O’Reilly C, et al. Guidelines for the management of severe traumatic brain injury. Neurosurgery. 2017;80:6–15. 27654000 10.1227/NEU.0000000000001432

[8] Shakur H. Effects of tranexamic acid on death, vascular occlusive events, and blood transfusion in trauma patients with significant haemorrhage (CRASH-2): a randomised, placebo-controlled trial. Lancet. 2010;376:23–32. 20554319 10.1016/S0140-6736(10)60835-5

[9] Crash T. Effects of tranexamic acid on death, disability, vascular occlusive events and other morbidities in patients with acute traumatic brain injury (CRASH-3): a randomised, placebo-controlled trial. Lancet. 2019;394:1713–23. 31623894 10.1016/S0140-6736(19)32233-0 PMC6853170

[10] Song J, Wu J, Zhong H, Chen W, Zheng J. Therapeutic efficacy of tranexamic acid on traumatic brain injury: a systematic review and meta-analysis. Scand J Trauma Resusc Emerg Med. 2024;32:18. 38454455 10.1186/s13049-024-01188-z PMC10921791

[11] Pulaski EJ, Reichel H, Voorhees AB. Effects of adrenoxyl on blood coagulation mechanism and vasomotor response. Exp Biol Med. 1949;70:504–7. 10.3181/00379727-70-16973 18116462

[12] Stovner J, Brennhovd I. The effect of ADRENOXYL® on blood loss during radical mastectomy. Acta Anaesthesiol Scand. 1961;5:33–7.

[13] Matsumoto Y, Hayashi T, Hayakawa Y, Shinbo M, Niiya K, Sakuragawa N. Carbazochrome sodium sulphonate (AC-17) decreases the accumulation of tissue-type plasminogen activator in culture medium of human umbilical vein endothelial cells. Blood Coagul Fibrinolysis. 1995;6:233–8. 7654937 10.1097/00001721-199505000-00006

[14] Sendo T, Itoh Y, Aki K, Oka M, Oishi R. Carbazochrome sodium sulfonate (AC-17) reverses endothelial barrier dysfunction through inhibition of phosphatidylinositol hydrolysis in cultured porcine endothelial cells. Naunyn Schmiedebergs Arch Pharmacol. 2003;368:175–80. 12928765 10.1007/s00210-003-0785-5

[15] Kinoshita K, Ishizaki Y, Yamamoto H, et al. De novo p.G696S mutation in COL4A1 causes intracranial calcification and late-onset cerebral hemorrhage: a case report and review of the literature. Eur J Med Genet. 2020;63:103825. 31857254 10.1016/j.ejmg.2019.103825

[16] De Rosa E. Treatment of massive upper gastrointestinal hemorrhage with carbazochrome salicylate. Int Surg. 1970;54:428–31. 5312690

[17] Basile M, Gidaro S, Pacella M, Biffignandi PM, Gidaro GS. Parenteral troxerutin and carbazochrome combination in the treatment of post-hemorrhoidectomy status: a randomized, double-blind, placebo-controlled, phase IV study. Curr Med Res Opin. 2001;17:256–61. 11922398

[18] Takeda K, Kawashima M, Masuda K, et al. A 65-year-old man with massive hemoptysis. Chest. 2023;164:e9–13. 37423707 10.1016/j.chest.2023.01.003

[19] Takahashi K, Sasaki T, Ueno N, et al. Carbazochrome sodium sulfonate is not effective for prevention of post-gastric endoscopic submucosal dissection bleeding: a retrospective study. Surg Endosc. 2022;36:7486–93. 35257213 10.1007/s00464-022-09171-4 PMC9485174

[20] Okazaki Y, Takada H, Okada I, Hasegawa E. Effect of Carbazochrome Sodium Sulfonate in addition to tranexamic acid in bleeding trauma patients. Cureus. 2022;14:e22018. 35282544 10.7759/cureus.22018 PMC8908800

[21] Luo Y, Releken Y, Yang D, Yue Y, Liu Z, Kang P. Effects of carbazochrome sodium sulfonate combined with tranexamic acid on hemostasis and inflammation during perioperative period of total hip arthroplasty: a randomized controlled trial. Orthop Traumatol Surg Res. 2022;108:103092. 34601160 10.1016/j.otsr.2021.103092

[22] Passali GC, De Corso E, Bastanza G, Di Gennaro L. An old drug for a new application: carbazochrome-sodium-sulfonate in HHT. J Clin Pharmacol. 2015;55:601–2. 25644784 10.1002/jcph.452

[23] Utsumi S, Ohki S, Shime N. Efficacy of tranexamic acid in adult isolated traumatic brain injury: a multicenter retrospective study. Am J Emerg Med. 2025;95:173–8. 40479948 10.1016/j.ajem.2025.05.030

[24] Yasunaga H. Updated information on the diagnosis procedure combination data. Ann Clin Epidemiol. 2024;6:106–10. 39726797 10.37737/ace.24015 PMC11668689

[25] Yamana H, Matsui H, Sasabuchi Y, Fushimi K, Yasunaga H. Categorized diagnoses and procedure records in an administrative database improved mortality prediction. J Clin Epidemiol. 2015;68:1028–35. 25596112 10.1016/j.jclinepi.2014.12.004

[26] Yamana H, Moriwaki M, Horiguchi H, Kodan M, Fushimi K, Yasunaga H. Validity of diagnoses, procedures, and laboratory data in Japanese administrative data. J Epidemiol. 2017;27:476–82. 28142051 10.1016/j.je.2016.09.009 PMC5602797

[27] Nil. Rating the severity of tissue damage: I the abbreviated scale. JAMA. 1971;215:277–80. 5107365 10.1001/jama.1971.03180150059012

[28] Clark DE, Black AW, Skavdahl DH, Hallagan LD. Open-access programs for injury categorization using ICD-9 or ICD-10. Inj Epidemiol. 2018;5:11. 29629480 10.1186/s40621-018-0149-8 PMC5890002

[29] Van Deynse H, Cools W, Depreitere B, et al. Quantifying injury severity for traumatic brain injury with routinely collected health data. Injury. 2022;53:11. 34702594 10.1016/j.injury.2021.10.013

[30] Osterwalder JJ. Could a regional trauma system in eastern Switzerland decrease the mortality of blunt polytrauma patients? A prospective cohort study. J Trauma. 2002;52:1030–6. 12045627 10.1097/00005373-200206000-00003

[31] Wada T, Yasunaga H, Yamana H, et al. Development and validation of a new ICD-10-based trauma mortality prediction scoring system using a Japanese national inpatient database. Inj Prev. 2016;23:263. 27597403 10.1136/injuryprev-2016-042106

[32] McKinley WI, Rowell SE, Mansour A, et al. Tranexamic acid, mortality, and intracranial hemorrhage type in moderate or severe traumatic brain injury. JAMA Surg. 2023;158:1222–4. 37755726 10.1001/jamasurg.2023.3848 PMC10535002

[33] Bossers SM, Loer SA, Bloemers FW, et al. Association between prehospital tranexamic acid administration and outcomes of severe traumatic brain injury. JAMA Neurol. 2021;78:338–45. 33284310 10.1001/jamaneurol.2020.4596 PMC7953275

[34] Utsumi S, Ohki S, Shime N. Epidemiology of moderate traumatic brain injury and factors associated with poor neurological outcome. J Neurosurg. 2024;141:430–5. 38552232 10.3171/2024.1.JNS232627

[35] Quan H, Li B, Couris CM, et al. Updating and validating the Charlson comorbidity index and score for risk adjustment in hospital discharge abstracts using data from 6 countries. Am J Epidemiol. 2011;173:676–82. 21330339 10.1093/aje/kwq433

[36] Barba C, Cavalli-Sforza T, Cutter J, Darnton-Hill I. Appropriate body-mass index for Asian populations and its implications for policy and intervention strategies. Lancet. 2004;363:157–63. 14726171 10.1016/S0140-6736(03)15268-3

[37] Nakajima M, Okada Y, Sonoo T, Goto T. Development and validation of a novel method for converting the Japan coma scale to Glasgow coma scale. J Epidemiol. 2023;33:531. 35851565 10.2188/jea.JE20220147 PMC10483104

[38] White IR, Royston P, Wood AM. Multiple imputation using chained equations: issues and guidance for practice. Stat Med. 2011;30:377–99. 21225900 10.1002/sim.4067

[39] Thomas LE, Li F, Pencina MJ. Overlap weighting: a propensity score method that mimics attributes of a randomized clinical trial. JAMA. 2020;323:2417–8. 32369102 10.1001/jama.2020.7819

[40] Li F, Morgan KL, Zaslavsky AM. Balancing covariates via propensity score weighting. J Am Stat Assoc. 2018;113:390–400.

[41] Nagasawa H, Omori K, Ota S, et al. Carbazochrome sodium sulfonate and tranexamic acid combination therapy to reduce blood transfusions after 24 h of injury: a retrospective study. Acute Med Surg. 2024;11:e961. 38715930 10.1002/ams2.961 PMC11074378

[42] Luo Y, Zhao X, Releken Y, Yang Z, Pei F, Kang P. Hemostatic and anti-inflammatory effects of carbazochrome sodium sulfonate in patients undergoing total knee arthroplasty: a randomized controlled trial. J Arthroplasty. 2020;35:61–8. 31471180 10.1016/j.arth.2019.07.045

[43] Alsaied MA, El-Sayed OS, Alqato S, Elettreby AM, Abo Elnaga AA. Optimizing blood management in arthroplasty: a meta-analysis of carbazochrome sodium sulfonate and tranexamic acid combination. J Orthop Surg Res. 2025;20:668. 40676674 10.1186/s13018-025-06038-x PMC12273061

[44] Sprigg N, Flaherty K, Appleton JP, et al. Tranexamic acid for hyperacute primary IntraCerebral Haemorrhage (TICH-2): an international randomised, placebo-controlled, phase 3 superiority trial. Lancet. 2018;391:2107–15. 29778325 10.1016/S0140-6736(18)31033-X PMC5976950

[45] Mayer SA, Brun NC, Begtrup K, et al. Efficacy and safety of recombinant activated factor VII for acute intracerebral hemorrhage. N Engl J Med. 2008;358:2127–37. 18480205 10.1056/NEJMoa0707534

[46] Kellie G. An account of the appearances observed in the dissection of two of three individuals presumed to have perished in the storm of the 3d, and whose bodies were discovered in the vicinity of Leith on the morning of the 4th, November 1821; with some reflections on the pathology of the brain: part I. Trans Med Chir Soc Edinb. 1824;1:84–122. 29583621 PMC5405298

[47] Monro A. Observations on the structure and functions of the nervous system. Lond Med J. 1783;4:113–35.

[48] Speth A, Dell’Orco A, Kleine JF, et al. Brain atrophy is associated with hematoma expansion in intracerebral hemorrhage, depending on coagulation status. J Clin Med. 2025;14:2227. 40217678 10.3390/jcm14072227 PMC11989702

[49] Zhang J, He M, Song Y, Xu J. Prognostic role of D-dimer level upon admission in patients with traumatic brain injury. Medicine Baltimore. 2018;97:e11774. 30075606 10.1097/MD.0000000000011774 PMC6081171

[50] Takahashi K, Iwama T, Tanaka K, et al. Risk factors for post-colorectal endoscopic submucosal dissection bleeding and efficacy of carbazochrome sodium sulfonate: a multicenter retrospective cohort study. Digestion. 2024;105:310–9. 38763127 10.1159/000539367 PMC11318496

