# Supplementary material for: Outcomes of combined carbazochrome sodium sulfonate plus tranexamic acid therapy versus tranexamic acid monotherapy in traumatic brain injury: a retrospective cohort study in Japan
Source: J Intensive Care. 2026 Jan 27;14:18. doi: 10.1186/s40560-026-00855-w (PMC12917989; doi:10.1186/s40560-026-00855-w)
Supplement: Supplementary file 1 — Supplementary Material 1. [file 40560_2026_855_MOESM1_ESM.docx]

Supplemental Table 1. ICD-10 codes and the names of traumatic brain injuries

| ICD-10 codes | Names of diagnosis |
| --- | --- |
| S061 | Traumatic cerebral edema |
| S062 | Diffuse brain injury |
| S063 | Focal brain hemorrhage |
| S064 | Epidural hemorrhage |
| S065 | Subdural hemorrhage |
| S066 | Subarachnoid hemorrhage |
| S068 | Other specified intracranial injuries |

Abbreviation: ICD-10, International Classification of Diseases 10th Revision.

Supplemental Table 2. ICD-10 codes of severe trauma with vascular injury and/or thoraco-abdominal organ injury

| Groups | ICD-10 codes |
| --- | --- |
| Neck | S15, S18, T04 |
| Thorax | S25, S26, S27, S28.1, T05, T06.5, T09.6 |
| Abdomen or pelvis | S35, S36, S37, S38.3, S39.6, T05.8, T06.5, T09.6 |
| Upper extremities | S45, S48, S55, S58, S65, S68, T11.4, T11.6 |
| Lower extremities | S75, S78, S85, S88, S95, S98, T13.4, T13.6 |
| Unspecified | T06.3 |

Abbreviation: ICD-10, International Classification of Diseases 10th Revision.

Supplemental Table 3. Neurosurgeries to be adjusted as possible confounder.

| K-code | Name of surgery |
| --- | --- |
| K145 | Burr hole ventricular drainage |
| K147 | Burr hole trephination |
| K147-2 | Intracranial pressure monitor insertion |
| K148 | Burr hole trephination and exploratory craniotomy (2 or more sites) |
| K1492 | Decompressive craniectomy (other) |
| K149-2 | Posterior fossa decompression |
| K160 | Craniotomy (Open surgery) |
| K163 | Subperiosteal hematoma evacuation |
| K164 | Craniotomy for hematoma evacuation |
| K1742 | Hydrocephalus surgery |

Note: K-code is the procedural code for surgery and procedure designated in medical billing system.

Supplemental Table 4. Baseline patient characteristics before and after propensity score overlap weighting

|  | Before weighting | | | | | After PS overlap weighting | | |
| --- | --- | --- | --- | --- | --- | --- | --- | --- |
|  | TXA only | | CSS plus TXA | | ASD | TXA only | CSS plus TXA | ASD |
| Variable | (n = 17,212) | | (n = 132,814) | |  |  | |  |
| Age, median (SD) | 71.7 | (17.2) | 73.9 | (15.6) | 0.13 | 72.4 (16.7) | 72.4 (16.6) | 0.00 |
| Male | 10584 | (61%) | 78748 | (59%) | 0.05 | (61%) | (61%) | 0.00 |
| Body mass index |  |  |  |  |  |  |  |  |
| <18.5 | 2774 | (16%) | 23906 | (18%) | 0.05 | (17%) | (17%) | 0.00 |
| 18.5–23 | 8029 | (47%) | 63521 | (48%) | 0.02 | (47%) | (47%) | 0.00 |
| 23–25 | 3081 | (18%) | 23049 | (17%) | 0.01 | (18%) | (18%) | 0.00 |
| >25 | 3328 | (19%) | 22338 | (17%) | 0.07 | (19%) | (19%) | 0.00 |
| Current/past smoker | 4938 | (29%) | 36675 | (28%) | 0.02 | (29%) | (29%) | 0.00 |
| Comorbidities |  |  |  |  |  |  |  |  |
| Myocardial infarction | 174 | (1.0%) | 1398 | (1.1%) | 0.00 | (1.0%) | (1.0%) | 0.00 |
| Congestive heart failure | 535 | (3%) | 5284 | (4%) | 0.05 | (3%) | (3%) | 0.00 |
| Peripheral vascular disease | 113 | (0.7%) | 984 | (0.7%) | 0.01 | (0.7%) | (0.7%) | 0.00 |
| Cerebrovascular disease | 1084 | (6%) | 10749 | (8%) | 0.07 | (7%) | (7%) | 0.00 |
| Dementia | 893 | (5%) | 9177 | (7%) | 0.07 | (6%) | (6%) | 0.00 |
| Chronic pulmonary disease | 215 | (1%) | 2052 | (2%) | 0.03 | (1%) | (1%) | 0.00 |
| Rheumatic disease | 77 | (0.5%) | 748 | (0.6%) | 0.02 | (0.5%) | (0.5%) | 0.00 |
| Peptic ulcer disease | 626 | (4%) | 6068 | (5%) | 0.05 | (4%) | (4%) | 0.00 |
| Mild liver disease | 332 | (2%) | 3360 | (3%) | 0.04 | (2%) | (2%) | 0.00 |
| Severe liver disease | 2180 | (13%) | 18525 | (14%) | 0.04 | (13%) | (13%) | 0.00 |
| Diabetes w/o complication | 323 | (2%) | 2428 | (2%) | 0.00 | (2%) | (2%) | 0.00 |
| Diabetes w/ complication | 225 | (1%) | 2694 | (2%) | 0.06 | (1%) | (1%) | 0.00 |
| Hemiplegia/paraplegia | 571 | (3%) | 4239 | (3%) | 0.01 | (3%) | (3%) | 0.00 |
| Renal disease | 475 | (3%) | 4328 | (3%) | 0.03 | (3%) | (3%) | 0.00 |
| Malignancy/lymphoma/leukemia | 20 | (0.1%) | 204 | (0.2%) | 0.01 | (0.1%) | (0.1%) | 0.00 |
| Metastatic solid tumor | 50 | (0.3%) | 528 | (0.4%) | 0.02 | (0.3%) | (0.3%) | 0.00 |
| AIDS/HIV | 3 | (0.0%) | 19 | (0.0%) | 0.00 | (0.0%) | (0.0%) | 0.00 |
| AIS of head, mean (SD) | 1.8 | (0.8) | 1.7 | (0.7) | 0.06 | 1.8 (0.8) | 1.8 (0.8) | 0.00 |
| ISS, mean (SD) | 4.4 | (4.1) | 3.9 | (3.3) | 0.14 | 4.2 (3.7) | 4.2 (4.1) | 0.00 |
| Details of head injury |  |  |  |  |  |  |  |  |
| Brain edema | 16 | (0.1%) | 85 | (0.1%) | 0.01 | (0.1%) | (0.1%) | 0.00 |
| DAI | 3309 | (19%) | 22853 | (17%) | 0.05 | (19%) | (19%) | 0.00 |
| Focal hematoma | 64 | (0.4%) | 482 | (0.4%) | 0.00 | (0.4%) | (0.4%) | 0.00 |
| AEDH | 85 | (0.5%) | 498 | (0.4%) | 0.02 | (0.5%) | (0.5%) | 0.00 |
| ASDH | 7747 | (45%) | 64684 | (49%) | 0.07 | (46%) | (46%) | 0.00 |
| Traumatic SAH | 6517 | (38%) | 45541 | (34%) | 0.07 | (37%) | (37%) | 0.00 |
| Others | 460 | (3%) | 4370 | (3%) | 0.04 | (3%) | (3%) | 0.00 |
| Japan Coma Scale |  |  |  |  |  |  |  |  |
| 0 (alert) | 4082 | (24%) | 37718 | (28%) | 0.11 | (25%) | (25%) | 0.00 |
| 1–3 (awake) | 6786 | (39%) | 59850 | (45%) | 0.11 | (41%) | (41%) | 0.00 |
| 1 | 2741 | (16%) | 25105 | (19%) | 0.08 | (17%) | (17%) | 0.00 |
| 2 | 2387 | (14%) | 19619 | (15%) | 0.03 | (14%) | (14%) | 0.00 |
| 3 | 1658 | (10%) | 15126 | (11%) | 0.06 | (10%) | (10%) | 0.00 |
| 10-30 (arousable) | 2424 | (14%) | 17128 | (13%) | 0.04 | (14%) | (14%) | 0.00 |
| 10 | 1516 | (9%) | 10650 | (8%) | 0.03 | (9%) | (9%) | 0.00 |
| 20 | 484 | (3%) | 3427 | (3%) | 0.01 | (3%) | (3%) | 0.00 |
| 30 | 424 | (2%) | 3051 | (2%) | 0.01 | (2%) | (2%) | 0.00 |
| 100–300  (unarousable) | 3920 | (23%) | 18118 | (14%) | 0.24 | (20%) | (20%) | 0.00 |
| 100 | 891 | (5%) | 4589 | (3%) | 0.09 | (5%) | (5%) | 0.00 |
| 200 | 1354 | (8%) | 7517 | (6%) | 0.09 | (7%) | (7%) | 0.00 |
| 300 | 1675 | (10%) | 6012 | (5%) | 0.20 | (8%) | (8%) | 0.00 |
| Treatments on admission day |  |  |  |  |  |  |  |  |
| Oxygen therapy | 4253 | (25%) | 31498 | (24%) | 0.02 | (25%) | (25%) | 0.00 |
| Intubation | 2861 | (17%) | 7886 | (6%) | 0.34 | (13%) | (13%) | 0.00 |
| Dialysis | 35 | (0.2%) | 386 | (0.3%) | 0.02 | (0.2%) | (0.2%) | 0.00 |
| Vasopressor |  |  |  |  |  |  |  |  |
| Adrenaline | 383 | (2%) | 1358 | (1%) | 0.10 | (2%) | (2%) | 0.00 |
| Noradrenaline | 1042 | (6%) | 2808 | (2%) | 0.20 | (5%) | (5%) | 0.00 |
| Vasopressin | 112 | (0.7%) | 200 | (0.2%) | 0.08 | (0.4%) | (0.4%) | 0.00 |
| Dopamine | 432 | (3%) | 2692 | (2%) | 0.03 | (2%) | (2%) | 0.00 |
| Dobutamine | 53 | (0.3%) | 225 | (0.2%) | 0.03 | (0.3%) | (0.3%) | 0.00 |
| FFP | 2100 | (12%) | 5999 | (5%) | 0.28 | (9%) | (9%) | 0.00 |
| Platelets | 625 | (4%) | 2529 | (2%) | 0.11 | (3%) | (3%) | 0.00 |
| Anticoagulant–antagonist |  |  |  |  |  |  |  |  |
| Prothrombin complex concentrate | 135 | (0.8%) | 587 | (0.4%) | 0.04 | (0.7%) | (0.7%) | 0.00 |
| Menatetrenone | 725 | (4%) | 6949 | (5%) | 0.05 | (4%) | (4%) | 0.00 |
| Idarucizumab | 17 | (0.1%) | 50 | (0.0%) | 0.02 | (0.1%) | (0.1%) | 0.00 |
| Fiscal year |  |  |  |  |  |  |  |  |
| 2010 | 391 | (2%) | 6426 | (5%) | 0.14 | (3%) | (3%) | 0.00 |
| 2011 | 661 | (4%) | 9735 | (7%) | 0.15 | (4%) | (4%) | 0.00 |
| 2012 | 748 | (4%) | 9979 | (8%) | 0.13 | (5%) | (5%) | 0.00 |
| 2013 | 841 | (5%) | 9837 | (7%) | 0.11 | (5%) | (5%) | 0.00 |
| 2014 | 1093 | (6%) | 11267 | (8%) | 0.08 | (7%) | (7%) | 0.00 |
| 2015 | 1126 | (7%) | 11161 | (8%) | 0.07 | (7%) | (7%) | 0.00 |
| 2016 | 1434 | (8%) | 12403 | (9%) | 0.04 | (9%) | (9%) | 0.00 |
| 2017 | 1434 | (8%) | 11956 | (9%) | 0.02 | (9%) | (9%) | 0.00 |
| 2018 | 1476 | (9%) | 10786 | (8%) | 0.02 | (9%) | (9%) | 0.00 |
| 2019 | 1722 | (10%) | 10184 | (8%) | 0.08 | (10%) | (10%) | 0.00 |
| 2020 | 1956 | (11%) | 10196 | (8%) | 0.13 | (11%) | (11%) | 0.00 |
| 2021 | 1988 | (12%) | 9564 | (7%) | 0.15 | (11%) | (11%) | 0.00 |
| 2022 | 2342 | (14%) | 9320 | (7%) | 0.22 | (12%) | (12%) | 0.00 |
| Season |  |  |  |  |  |  |  |  |
| Dec–Feb | 4557 | (26%) | 34866 | (26%) | 0.01 | (26%) | (26%) | 0.00 |
| Mar–May | 3899 | (23%) | 30723 | (23%) | 0.01 | (23%) | (23%) | 0.00 |
| Jun–Aug | 4012 | (23%) | 31151 | (23%) | 0.00 | (23%) | (23%) | 0.00 |
| Sep–Nov | 4744 | (28%) | 36074 | (27%) | 0.01 | (27%) | (27%) | 0.00 |
| TBI related hospital volume, per year |  |  |  |  |  |  |  |  |
| < 31 | 11023 | (64%) | 75990 | (57%) | 0.14 | (62%) | (62%) | 0.00 |
| 31–60 | 4783 | (28%) | 42284 | (32%) | 0.09 | (29%) | (29%) | 0.00 |
| >60 | 1406 | (8%) | 14540 | (11%) | 0.10 | (9%) | (9%) | 0.00 |
| Ambulance use | 15254 | (89%) | 108946 | (82%) | 0.19 | (87%) | (87%) | 0.00 |
| Admission on weekends or at night | 1730 | (10%) | 19601 | (15%) | 0.14 | (11%) | (11%) | 0.00 |
| Admission ward |  |  |  |  |  |  |  |  |
| General ward | 6698 | (39%) | 70868 | (53%) | 0.29 | (42%) | (42%) | 0.00 |
| ICU | 4757 | (28%) | 22414 | (17%) | 0.26 | (24%) | (24%) | 0.00 |
| HDU | 5714 | (33%) | 39083 | (29%) | 0.08 | (33%) | (33%) | 0.00 |
| SCU | 43 | (0.3%) | 449 | (0.3%) | 0.02 | (0.3%) | (0.3%) | 0.00 |
| Teaching hospital | 15664 | (91%) | 112120 | (84%) | 0.20 | (90%) | (90%) | 0.00 |
| University hospital | 3054 | (18%) | 10386 | (8%) | 0.30 | (15%) | (15%) | 0.00 |

Reported as n (%) or (%), unless indicated otherwise. The absolute standardized difference (ASD) was calculated after multiple imputations.

Abbreviations: SD, standard deviation; w/, with; w/o, without; AIDS, acquired immunodeficiency syndrome; HIV, human immunodeficiency virus; AIS, Abbreviated Injury Scale; ISS, injury severity score; DAI, diffuse axonal injury; AEDH, acute epidural hemorrhage; ASDH, acute subdural hemorrhage; SAH, sub arachnoid hemorrhage; FFP, fresh frozen plasma; TBI, traumatic brain injury; ICU, intensive care unit; HDU, high-dependency unit; SCU, stroke care unit.

Supplemental Table 5. Baseline patient characteristics before and after propensity score overlap weighting; complete cases analysis

|  | Before weighting | | | | | After PS overlap weighting | | |
| --- | --- | --- | --- | --- | --- | --- | --- | --- |
|  | TXA only | | CSS plus TXA | | ASD | TXA only | CSS plus TXA | ASD |
| Variable | (n = 17,212) | | (n = 132,814) | |  |  | |  |
| Age, median (SD) | 72 | (17.2) | 74 | (15.6) | 0.12 | 72.6 (16.8) | 72.6 (16.6) | 0.00 |
| Male | 7058 | (59%) | 56119 | (57%) | 0.05 | (59%) | (59%) | 0.00 |
| Body mass index |  |  |  |  |  |  |  |  |
| <18.5 | 1926 | (16%) | 17612 | (18%) | 0.05 | (17%) | (17%) | 0.00 |
| 18.5–23 | 5566 | (47%) | 47325 | (48%) | 0.03 | (47%) | (47%) | 0.00 |
| 23–25 | 2099 | (18%) | 16914 | (17%) | 0.01 | (18%) | (18%) | 0.00 |
| >25 | 2303 | (19%) | 16357 | (17%) | 0.07 | (19%) | (19%) | 0.00 |
| Current/past smoker | 3435 | (29%) | 27118 | (28%) | 0.03 | (29%) | (29%) | 0.00 |
| Comorbidities |  |  |  |  |  |  |  |  |
| Myocardial infarction | 134 | (1.1%) | 1103 | (1.1%) | 0.00 | (1.1%) | (1.1%) | 0.00 |
| Congestive heart failure | 409 | (3%) | 4171 | (4%) | 0.04 | (4%) | (4%) | 0.00 |
| Peripheral vascular disease | 87 | (0.7%) | 782 | (0.8%) | 0.01 | (0.8%) | (0.8%) | 0.00 |
| Cerebrovascular disease | 797 | (7%) | 8238 | (8%) | 0.06 | (7%) | (7%) | 0.00 |
| Dementia | 658 | (6%) | 7000 | (7%) | 0.07 | (6%) | (6%) | 0.00 |
| Chronic pulmonary disease | 158 | (1.3%) | 1590 | (1.6%) | 0.02 | (1.4%) | (1.4%) | 0.00 |
| Rheumatic disease | 62 | (0.5%) | 600 | (0.6%) | 0.01 | (0.5%) | (0.5%) | 0.00 |
| Peptic ulcer disease | 423 | (4%) | 4501 | (5%) | 0.05 | (4%) | (4%) | 0.00 |
| Mild liver disease | 238 | (2%) | 2578 | (3%) | 0.04 | (2%) | (2%) | 0.00 |
| Severe liver disease | 1606 | (14%) | 14377 | (15%) | 0.03 | (14%) | (14%) | 0.00 |
| Diabetes w/o complication | 241 | (2%) | 1943 | (2%) | 0.00 | (2%) | (2%) | 0.00 |
| Diabetes w/ complication | 164 | (1%) | 2104 | (2%) | 0.06 | (2%) | (2%) | 0.00 |
| Hemiplegia/paraplegia | 418 | (4%) | 3310 | (3%) | 0.01 | (4%) | (4%) | 0.00 |
| Renal disease | 373 | (3%) | 3428 | (3%) | 0.02 | (3%) | (3%) | 0.00 |
| Malignancy/lymphoma/leukemia | 14 | (0.1%) | 150 | (0.2%) | 0.01 | (0.1%) | (0.1%) | 0.00 |
| Metastatic solid tumor | 42 | (0.4%) | 411 | (0.4%) | 0.01 | (0.4%) | (0.4%) | 0.00 |
| AIDS/HIV | 3 | (0.0%) | 11 | (0.0%) | 0.01 | (0.0%) | (0.0%) | 0.00 |
| AIS of head, mean (SD) | 1.8 | (0.8) | 1.7 | (0.7) | 0.05 | 1.8 (0.8) | 1.8 (0.8) | 0.00 |
| ISS, mean (SD) | 4.3 | (4.0) | 3.8 | (3.2) | 0.13 | 4.1 (3.7) | 4.1 (4.0) | 0.00 |
| Details of head injury |  |  |  |  |  |  |  |  |
| Brain edema | 11 | (0.1%) | 58 | (0.1%) | 0.01 | (0.1%) | (0.1%) | 0.00 |
| DAI | 2224 | (19%) | 16600 | (17%) | 0.05 | (18%) | (18%) | 0.00 |
| Focal hematoma | 42 | (0.4%) | 351 | (0.4%) | 0.00 | (0.4%) | (0.4%) | 0.00 |
| AEDH | 58 | (0.5%) | 363 | (0.4%) | 0.02 | (0.4%) | (0.4%) | 0.00 |
| ASDH | 5353 | (45%) | 47880 | (49%) | 0.08 | (46%) | (46%) | 0.00 |
| Traumatic SAH | 4539 | (38%) | 33974 | (35%) | 0.07 | (38%) | (38%) | 0.00 |
| Others | 315 | (3%) | 3125 | (3%) | 0.03 | (3%) | (3%) | 0.00 |
| Japan Coma Scale |  |  |  |  |  |  |  |  |
| 0 (alert) | 3019 | (25%) | 29141 | (30%) | 0.10 | (26%) | (26%) | 0.00 |
| 1–3 (awake) | 4935 | (41%) | 44849 | (46%) | 0.08 | (43%) | (43%) | 0.00 |
| 1 | 2064 | (17%) | 19334 | (20%) | 0.06 | (18%) | (18%) | 0.00 |
| 2 | 1740 | (15%) | 14696 | (15%) | 0.01 | (15%) | (15%) | 0.00 |
| 3 | 1131 | (10%) | 10819 | (11%) | 0.05 | (10%) | (10%) | 0.00 |
| 10-30 (arousable) | 1676 | (14%) | 12514 | (13%) | 0.04 | (14%) | (14%) | 0.00 |
| 10 | 1069 | (9%) | 7913 | (8%) | 0.03 | (9%) | (9%) | 0.00 |
| 20 | 321 | (3%) | 2453 | (3%) | 0.01 | (3%) | (3%) | 0.00 |
| 30 | 286 | (2%) | 2148 | (2%) | 0.02 | (2%) | (2%) | 0.00 |
| 100–300  (unarousable) | 2264 | (19%) | 11704 | (12%) | 0.20 | (17%) | (17%) | 0.00 |
| 100 | 573 | (5%) | 3097 | (3%) | 0.09 | (4%) | (4%) | 0.00 |
| 200 | 839 | (7%) | 4985 | (5%) | 0.08 | (7%) | (7%) | 0.00 |
| 300 | 852 | (7%) | 3622 | (4%) | 0.15 | (6%) | (6%) | 0.00 |
| Treatments on admission day |  |  |  |  |  |  |  |  |
| Oxygen therapy | 2983 | (25%) | 23226 | (24%) | 0.03 | (25%) | (25%) | 0.00 |
| Intubation | 1604 | (13%) | 5034 | (5%) | 0.29 | (11%) | (11%) | 0.00 |
| Dialysis | 23 | (0.2%) | 314 | (0.3%) | 0.03 | (0.2%) | (0.2%) | 0.00 |
| Vasopressor |  |  |  |  |  |  |  |  |
| Adrenaline | 177 | (1.5%) | 866 | (0.9%) | 0.06 | (1.2%) | (1.2%) | 0.00 |
| Noradrenaline | 545 | (5%) | 1787 | (2%) | 0.16 | (4%) | (4%) | 0.00 |
| Vasopressin | 54 | (0.5%) | 115 | (0.1%) | 0.06 | (0.3%) | (0.3%) | 0.00 |
| Dopamine | 262 | (2%) | 1787 | (2%) | 0.03 | (2%) | (2%) | 0.00 |
| Dobutamine | 35 | (0.3%) | 154 | (0.2%) | 0.03 | (0.3%) | (0.3%) | 0.00 |
| FFP | 1239 | (10%) | 4162 | (4%) | 0.24 | (8%) | (8%) | 0.00 |
| Platelets | 413 | (3%) | 1800 | (2%) | 0.10 | (3%) | (3%) | 0.00 |
| Anticoagulant-antagonist |  |  |  |  |  |  |  |  |
| Prothrombin complex concentrate | 110 | (0.9%) | 450 | (0.5%) | 0.06 | (0.9%) | (0.9%) | 0.00 |
| Menatetrenone | 499 | (4%) | 5193 | (5%) | 0.05 | (4%) | (4%) | 0.00 |
| Idarucizumab | 11 | (0.1%) | 36 | (0.0%) | 0.02 | (0.1%) | (0.1%) | 0.00 |
| Fiscal year |  |  |  |  |  |  |  |  |
| 2010 | 261 | (2%) | 4334 | (4%) | 0.12 | (2%) | (2%) | 0.00 |
| 2011 | 421 | (4%) | 6897 | (7%) | 0.16 | (4%) | (4%) | 0.00 |
| 2012 | 480 | (4%) | 7157 | (7%) | 0.14 | (4%) | (4%) | 0.00 |
| 2013 | 534 | (4%) | 7215 | (7%) | 0.12 | (5%) | (5%) | 0.00 |
| 2014 | 751 | (6%) | 8396 | (9%) | 0.09 | (7%) | (7%) | 0.00 |
| 2015 | 808 | (7%) | 8473 | (9%) | 0.07 | (7%) | (7%) | 0.00 |
| 2016 | 1015 | (9%) | 9435 | (10%) | 0.04 | (9%) | (9%) | 0.00 |
| 2017 | 1022 | (9%) | 9035 | (9%) | 0.02 | (9%) | (9%) | 0.00 |
| 2018 | 1025 | (9%) | 8038 | (8%) | 0.02 | (9%) | (9%) | 0.00 |
| 2019 | 1228 | (10%) | 7731 | (8%) | 0.09 | (10%) | (10%) | 0.00 |
| 2020 | 1378 | (12%) | 7655 | (8%) | 0.13 | (11%) | (11%) | 0.00 |
| 2021 | 1366 | (11%) | 7121 | (7%) | 0.15 | (11%) | (11%) | 0.00 |
| 2022 | 1605 | (13%) | 6721 | (7%) | 0.22 | (12%) | (12%) | 0.00 |
| Season |  |  |  |  |  |  |  |  |
| Dec–Feb | 3139 | (26%) | 25729 | (26%) | 0.00 | (26%) | (26%) | 0.00 |
| Mar–May | 2673 | (22%) | 22528 | (23%) | 0.01 | (23%) | (23%) | 0.00 |
| Jun–Aug | 2772 | (23%) | 23181 | (24%) | 0.01 | (23%) | (23%) | 0.00 |
| Sep–Nov | 3310 | (28%) | 26770 | (27%) | 0.01 | (28%) | (28%) | 0.00 |
| TBI related hospital volume, per year |  |  |  |  |  |  |  |  |
| < 31 | 7527 | (63%) | 55905 | (57%) | 0.13 | (62%) | (62%) | 0.00 |
| 31–60 | 3292 | (28%) | 31229 | (32%) | 0.09 | (29%) | (29%) | 0.00 |
| >60 | 1075 | (9%) | 11074 | (11%) | 0.07 | (10%) | (10%) | 0.00 |
| Ambulance use | 10408 | (88%) | 79655 | (81%) | 0.18 | (86%) | (86%) | 0.00 |
| Admission on weekends or at night | 1250 | (11%) | 14271 | (15%) | 0.12 | (11%) | (11%) | 0.00 |
| Admission ward |  |  |  |  |  |  |  |  |
| General ward | 4845 | (41%) | 52563 | (54%) | 0.26 | (44%) | (44%) | 0.00 |
| ICU | 3053 | (26%) | 16069 | (16%) | 0.23 | (23%) | (23%) | 0.00 |
| HDU | 3959 | (33%) | 29227 | (30%) | 0.08 | (33%) | (33%) | 0.00 |
| SCU | 37 | (0.3%) | 349 | (0.4%) | 0.01 | (0.3%) | (0.3%) | 0.00 |
| Teaching hospital | 10653 | (90%) | 81571 | (83%) | 0.19 | (88%) | (88%) | 0.00 |
| University hospital | 1957 | (16%) | 6587 | (7%) | 0.31 | (14%) | (14%) | 0.00 |

Reported as n (%) or (%), unless indicated otherwise.

Abbreviations: SD, standard deviation; w/, with; w/o, without; AIDS, acquired immunodeficiency syndrome; HIV, human immunodeficiency virus; AIS, Abbreviated Injury Scale; ISS, injury severity score; DAI, diffuse axonal injury; AEDH, acute epidural hemorrhage; ASDH, acute subdural hemorrhage; SAH, sub arachnoid hemorrhage; FFP, fresh frozen plasma; TBI, traumatic brain injury; ICU, intensive care unit; HDU, high-dependency unit; SCU, stroke care unit.

Supplemental Table 6. Baseline patient characteristics before and after propensity score overlap weighting; analysis with further adjustment for potential confounders (intracranial pressure monitoring, neurosurgery, and administration of antihypertensive agents and osmotic agents on day 1)

|  | Before weighting | | | | | After PS overlap weighting | | |
| --- | --- | --- | --- | --- | --- | --- | --- | --- |
|  | TXA only | | CSS plus TXA | | ASD | TXA only | CSS plus TXA | ASD |
| Variable | (n = 17,212) | | (n = 132,814) | |  |  | |  |
| Age, median (SD) | 71.7 | (17.2) | 73.9 | (15.6) | 0.13 | 72.4(16.7) | 72.4(16.7) | 0.00 |
| Male | 10584 | (1%) | 78748 | (1%) | 0.05 | (1%) | (1%) | 0.00 |
| Body mass index |  |  |  |  |  |  |  |  |
| <18.5 | 2781 | (16%) | 23923 | (18%) | 0.05 | (17%) | (17%) | 0.00 |
| 18.5–23 | 8020 | (47%) | 63499 | (48%) | 0.02 | (47%) | (47%) | 0.00 |
| 23–25 | 3090 | (18%) | 23037 | (17%) | 0.02 | (18%) | (18%) | 0.00 |
| >25 | 3321 | (19%) | 22355 | (17%) | 0.06 | (19%) | (19%) | 0.00 |
| Current/past smoker | 4938 | (29%) | 36666 | (28%) | 0.02 | (29%) | (29%) | 0.00 |
| Comorbidities |  |  |  |  |  |  |  |  |
| Myocardial infarction | 174 | (1.0%) | 1398 | (1.1%) | 0.00 | (1.0%) | (1.0%) | 0.00 |
| Congestive heart failure | 535 | (3%) | 5284 | (4%) | 0.05 | (3%) | (3%) | 0.00 |
| Peripheral vascular disease | 113 | (0.7%) | 984 | (0.7%) | 0.01 | (0.7%) | (0.7%) | 0.00 |
| Cerebrovascular disease | 1084 | (6%) | 10749 | (8%) | 0.07 | (7%) | (7%) | 0.00 |
| Dementia | 893 | (5%) | 9177 | (7%) | 0.07 | (6%) | (6%) | 0.00 |
| Chronic pulmonary disease | 215 | (1%) | 2052 | (2%) | 0.03 | (1%) | (1%) | 0.00 |
| Rheumatic disease | 77 | (0.5%) | 748 | (0.6%) | 0.02 | (0.5%) | (0.5%) | 0.00 |
| Peptic ulcer disease | 626 | (4%) | 6068 | (5%) | 0.05 | (4%) | (4%) | 0.00 |
| Mild liver disease | 332 | (2%) | 3360 | (3%) | 0.04 | (2%) | (2%) | 0.00 |
| Severe liver disease | 2180 | (13%) | 18525 | (14%) | 0.04 | (13%) | (13%) | 0.00 |
| Diabetes w/o complication | 323 | (2%) | 2428 | (2%) | 0.00 | (2%) | (2%) | 0.00 |
| Diabetes w/ complication | 225 | (1%) | 2694 | (2%) | 0.06 | (1%) | (1%) | 0.00 |
| Hemiplegia/paraplegia | 571 | (3%) | 4239 | (3%) | 0.01 | (3%) | (3%) | 0.00 |
| Renal disease | 475 | (3%) | 4328 | (3%) | 0.03 | (3%) | (3%) | 0.00 |
| Malignancy/lymphoma/leukemia | 20 | (0.1%) | 204 | (0.2%) | 0.01 | (0.1%) | (0.1%) | 0.00 |
| Metastatic solid tumor | 50 | (0.3%) | 528 | (0.4%) | 0.02 | (0.3%) | (0.3%) | 0.00 |
| AIDS/HIV | 3 | (0.0%) | 19 | (0.0%) | 0.00 | (0.0%) | (0.0%) | 0.00 |
| AIS of head, mean (SD) | 1.8 | -(0.8) | 1.7 | -(0.7) | 0.06 | 1.8(0.8) | 1.8(0.8) | 0.00 |
| ISS, mean (SD) | 4.4 | -(4.1) | 3.9 | -(3.3) | 0.14 | 4.2(3.7) | 4.2(4.0) | 0.00 |
| Details of head injury |  |  |  |  |  |  |  |  |
| Brain edema | 16 | (0.1%) | 85 | (0.1%) | 0.01 | (0.1%) | (0.1%) | 0.00 |
| DAI | 3309 | (19%) | 22853 | (17%) | 0.05 | (19%) | (19%) | 0.00 |
| Focal hematoma | 64 | (0.4%) | 482 | (0.4%) | 0.00 | (0.4%) | (0.4%) | 0.00 |
| AEDH | 85 | (0.5%) | 498 | (0.4%) | 0.02 | (0.5%) | (0.5%) | 0.00 |
| ASDH | 7747 | (45%) | 64684 | (49%) | 0.07 | (46%) | (46%) | 0.00 |
| Traumatic SAH | 6517 | (38%) | 45541 | (34%) | 0.07 | (37%) | (37%) | 0.00 |
| Others | 460 | (3%) | 4370 | (3%) | 0.04 | (3%) | (3%) | 0.00 |
| Japan Coma Scale |  |  |  |  |  |  |  |  |
| 0 (alert) | 4082 | (24%) | 37718 | (28%) | 0.11 | (25%) | (25%) | 0.00 |
| 1–3 (awake) | 6786 | (39%) | 59850 | (45%) | 0.11 | (41%) | (41%) | 0.00 |
| 1 | 2741 | (16%) | 25105 | (19%) | 0.08 | (17%) | (17%) | 0.00 |
| 2 | 2387 | (14%) | 19619 | (15%) | 0.03 | (14%) | (14%) | 0.00 |
| 3 | 1658 | (10%) | 15126 | (11%) | 0.06 | (10%) | (10%) | 0.00 |
| 10-30 (arousable) | 2424 | (14%) | 17128 | (13%) | 0.04 | (14%) | (14%) | 0.00 |
| 10 | 1516 | (9%) | 10650 | (8%) | 0.03 | (9%) | (9%) | 0.00 |
| 20 | 484 | (3%) | 3427 | (3%) | 0.01 | (3%) | (3%) | 0.00 |
| 30 | 424 | (2%) | 3051 | (2%) | 0.01 | (2%) | (2%) | 0.00 |
| 100–300  (unarousable) | 3920 | (23%) | 18118 | (14%) | 0.24 | (20%) | (20%) | 0.00 |
| 100 | 891 | (5%) | 4589 | (3%) | 0.09 | (5%) | (5%) | 0.00 |
| 200 | 1354 | (8%) | 7517 | (6%) | 0.09 | (7%) | (7%) | 0.00 |
| 300 | 1675 | (10%) | 6012 | (5%) | 0.20 | (8%) | (8%) | 0.00 |
| Treatments on admission day |  |  |  |  |  |  |  |  |
| Oxygen therapy | 4253 | (25%) | 31498 | (24%) | 0.02 | (25%) | (25%) | 0.00 |
| Intubation | 2861 | (17%) | 7886 | (6%) | 0.34 | (13%) | (13%) | 0.00 |
| Dialysis | 35 | (0.2%) | 386 | (0.3%) | 0.02 | (0.2%) | (0.2%) | 0.00 |
| Vasopressor |  |  |  |  |  |  |  |  |
| Adrenaline | 383 | (2%) | 1358 | (1%) | 0.10 | (2%) | (2%) | 0.00 |
| Noradrenaline | 1042 | (6%) | 2808 | (2%) | 0.20 | (5%) | (5%) | 0.00 |
| Vasopressin | 112 | (0.7%) | 200 | (0.2%) | 0.08 | (0.4%) | (0.4%) | 0.00 |
| Dopamine | 432 | (3%) | 2692 | (2%) | 0.03 | (2%) | (2%) | 0.00 |
| Dobutamine | 53 | (0.3%) | 225 | (0.2%) | 0.03 | (0.3%) | (0.3%) | 0.00 |
| FFP | 2100 | (0.1%) | 5999 | (0.0%) | 0.28 | (0.1%) | (0.1%) | 0.00 |
| Platelets | 625 | (0.0%) | 2529 | (0.0%) | 0.11 | (0.0%) | (0.0%) | 0.00 |
| Anticoagulant-antagonist |  |  |  |  |  |  |  |  |
| Prothrombin complex concentrate | 135 | (0.0%) | 587 | (0.0%) | 0.04 | (0.0%) | (0.0%) | 0.00 |
| Menatetrenone | 725 | (0.0%) | 6949 | (0.1%) | 0.05 | (0.0%) | (0.0%) | 0.00 |
| Idarucizumab | 17 | (0.0%) | 50 | (0.0%) | 0.02 | (0.0%) | (0.0%) | 0.00 |
| (Further adjustments) |  |  |  |  |  |  |  |  |
| Neurosurgery | 2284 | (13%) | 13764 | (10%) | 0.09 | (12%) | (12%) | 0.00 |
| ICP monitoring | 99 | (0.6%) | 74 | (0.1%) | 0.09 | (0.3%) | (0.3%) | 0.00 |
| Glycerol | 1574 | (9%) | 17148 | (13%) | 0.12 | (9%) | (9%) | 0.00 |
| Mannitol | 1663 | (10%) | 8847 | (7%) | 0.11 | (9%) | (9%) | 0.00 |
| Diltiazem | 255 | (1%) | 3929 | (3%) | 0.10 | (2%) | (2%) | 0.00 |
| Nicardipine | 6725 | (39%) | 49819 | (38%) | 0.03 | (39%) | (39%) | 0.00 |
| Verapamil | 64 | (0.4%) | 546 | (0.4%) | 0.01 | (0.4%) | (0.4%) | 0.00 |
| Fiscal year |  |  |  |  |  |  |  |  |
| 2010 | 391 | (2%) | 6426 | (5%) | 0.14 | (3%) | (3%) | 0.00 |
| 2011 | 661 | (4%) | 9735 | (7%) | 0.15 | (4%) | (4%) | 0.00 |
| 2012 | 748 | (4%) | 9979 | (8%) | 0.13 | (5%) | (5%) | 0.00 |
| 2013 | 841 | (5%) | 9837 | (7%) | 0.11 | (5%) | (5%) | 0.00 |
| 2014 | 1093 | (6%) | 11267 | (8%) | 0.08 | (7%) | (7%) | 0.00 |
| 2015 | 1126 | (7%) | 11161 | (8%) | 0.07 | (7%) | (7%) | 0.00 |
| 2016 | 1434 | (8%) | 12403 | (9%) | 0.04 | (9%) | (9%) | 0.00 |
| 2017 | 1434 | (8%) | 11956 | (9%) | 0.02 | (9%) | (9%) | 0.00 |
| 2018 | 1476 | (9%) | 10786 | (8%) | 0.02 | (9%) | (9%) | 0.00 |
| 2019 | 1722 | (10%) | 10184 | (8%) | 0.08 | (10%) | (10%) | 0.00 |
| 2020 | 1956 | (11%) | 10196 | (8%) | 0.13 | (11%) | (11%) | 0.00 |
| 2021 | 1988 | (12%) | 9564 | (7%) | 0.15 | (11%) | (11%) | 0.00 |
| 2022 | 2342 | (14%) | 9320 | (7%) | 0.22 | (12%) | (12%) | 0.00 |
| Season |  |  |  |  |  |  |  |  |
| Dec–Feb | 4557 | (26%) | 34866 | (26%) | 0.01 | (26%) | (26%) | 0.00 |
| Mar–May | 3899 | (23%) | 30723 | (23%) | 0.01 | (23%) | (23%) | 0.00 |
| Jun–Aug | 4012 | (23%) | 31151 | (23%) | 0.00 | (23%) | (23%) | 0.00 |
| Sep–Nov | 4744 | (28%) | 36074 | (27%) | 0.01 | (27%) | (27%) | 0.00 |
| TBI related hospital volume, per year |  |  |  |  |  |  |  |  |
| < 31 | 11023 | (64%) | 75990 | (57%) | 0.14 | (62%) | (62%) | 0.00 |
| 31–60 | 4783 | (28%) | 42284 | (32%) | 0.09 | (29%) | (29%) | 0.00 |
| >60 | 1406 | (8%) | 14540 | (11%) | 0.10 | (9%) | (9%) | 0.00 |
| Ambulance use | 15254 | (89%) | 108945 | (82%) | 0.19 | (87%) | (87%) | 0.00 |
| Admission on weekends or at night | 1730 | (10%) | 19601 | (15%) | 0.14 | (11%) | (11%) | 0.00 |
| Admission ward |  |  |  |  |  |  |  |  |
| General ward | 6698 | (39%) | 70868 | (53%) | 0.29 | (43%) | (43%) | 0.00 |
| ICU | 4757 | (28%) | 22414 | (17%) | 0.26 | (24%) | (24%) | 0.00 |
| HDU | 5714 | (33%) | 39083 | (29%) | 0.08 | (33%) | (33%) | 0.00 |
| SCU | 43 | (0.3%) | 449 | (0.3%) | 0.02 | (0.3%) | (0.3%) | 0.00 |
| Teaching hospital | 15664 | (91%) | 112120 | (84%) | 0.20 | (90%) | (90%) | 0.00 |
| University hospital | 3054 | (18%) | 10386 | (8%) | 0.30 | (15%) | (15%) | 0.00 |

Reported as n (%) or (%), unless indicated otherwise. The absolute standardized difference (ASD) was calculated after multiple imputations.

Abbreviations: SD, standard deviation; w/, with; w/o, without; AIDS, acquired immunodeficiency syndrome; HIV, human immunodeficiency virus; AIS, Abbreviated Injury Scale; ISS, injury severity score; DAI, diffuse axonal injury; AEDH, acute epidural hemorrhage; ASDH, acute subdural hemorrhage; SAH, sub arachnoid hemorrhage; FFP, fresh frozen plasma; TBI, traumatic brain injury; ICU, intensive care unit; HDU, high-dependency unit; SCU, stroke care unit.
